# Anthocyanins as Key Phytochemicals Acting for the Prevention of Metabolic Diseases: An Overview

**DOI:** 10.3390/molecules27134254

**Published:** 2022-07-01

**Authors:** Madalina Nistor, Roxana Pop, Adela Daescu, Adela Pintea, Carmen Socaciu, Dumitrita Rugina

**Affiliations:** 1 Department of Agricultural Egineering Sciences, University of Agricultural Sciences and Veterinary Medicine, Manastur Street 3-5, 400372 Cluj-Napoca, Romania; nistor.madalina@usamvcluj.ro (M.N.); contact@usamvcluj.ro (R.P.); adela.daescu@usamvcluj.ro (A.D.); carmen.socaciu@usamvcluj.ro (C.S.); 2 Department of Veterinary Medicine, University of Agricultural Sciences and Veterinary Medicine, Manastur Street 3-5, 400372 Cluj-Napoca, Romania; apintea@usamvcluj.ro

**Keywords:** absorption, anthocyanins, bioavailability, cancer, cardiovascular diseases, diabetes, inflammatory disorders, neuropathologies, oxidative stress

## Abstract

Anthocyanins are water-soluble pigments present in fruits and vegetables, which render them an extensive range of colors. They have a wide distribution in the human diet, are innocuous, and, based on numerous studies, have supposed preventive and therapeutical benefits against chronic affections such as inflammatory, neurological, cardiovascular, digestive disorders, diabetes, and cancer, mostly due to their antioxidant action. Despite their great potential as pharmaceutical applications, they have a rather limited use because of their rather low stability to environmental variations. Their absorption was noticed to occur best in the stomach and small intestine, but the pH fluctuation of the digestive system impacts their rapid degradation. Urine excretion and tissue distribution also occur at low rates. The aim of this review is to highlight the chemical characteristics of anthocyanins and emphasize their weaknesses regarding bioavailability. It also targets to deliver an update on the recent advances in the involvement of anthocyanins in different pathologies with a focus on in vivo, in vitro, animal, and human clinical trials.

## 1. Introduction

Anthocyanins (ANs) are natural components that give beautiful colors of blue, purple, pink and red to leaves, fruits, and flowers, and are responsible for a plethora of health beneficial functions as dietary antioxidants, that can fight free radicals which raise the risk of chronic diseases onset such as: neuronal disorders, inflammatory conditions, diabetes, obesity, cardiovascular diseases and cancer [1]. More than one thousand ANs have been identified in vegetal sources and over 600 are found in natural foods of vegetal sources [2].

ANs are plant water-soluble pigments that are synthesized in the cytoplasm and are stored in high concentrations in the vacuoles. [3,4,5]. In plants, ANs seem to facilitate pollination and seed dispersion, provide foliar protection against biotic and abiotic stress conditions, or act as sunscreen protection [1,6].

Over time, it was accepted that the daily consumption of fruits and vegetables prevents several chronic diseases and prolongs the healthy condition of humans. ANs are one of the main classes of nutraceuticals relevant to well-being due to their antioxidant, anti-inflammatory, antitumoral, and antimicrobial properties [7].

In addition to their importance as human and animal valuable food resources, they have another application in the food industry as natural colorants [8]. One such example is E163, a commercial food additive obtained from grape skin, rich in ANs, and used to color jams, sweets, or beverages. The advantage of consuming ANs is that they are not toxic, even in higher doses. Apart from their health benefits, they have value-added properties due to their antioxidant characteristic or an increasing appealing aspect in food [9,10].

With their vast distribution and low dietary toxicity, ANs are superior compared to other flavonoids in terms of human consumption, with an estimated intake of 200 mg/day [11,12]. However, there is one downfall to them, namely, their color and function stability is highly labile and strongly affected by environmental factors such as pH, temperature, light exposure, oxygen, copigmentation, enzymes, solvents, and chemical structure [13].

This aspect is particularly reflected in their structural fate, once they are ingested and subjected to travel through diverse and extreme changes of environmental conditions, which impacts their final capacity to exert their beneficial functions [7]. The digestion of ANs starts in the oral cavity. The majority of ANs reach the stomach where, due to optimal pH conditions, can partly be absorbed directly in the blood plasma and delivered to the liver, and a higher amount reaches the small intestine, where they suffer structural modifications and produce degradation metabolites, that are also uptaken by the liver and furtherly distributed to other tissues [14].

The purpose of this review is to provide an overview of the chemical properties of ANs and their therapeutical advances, with emphasis on the most recent advances in in vivo and in vitro biological studies with additional clustering of the methods used for their extraction and qualitative/quantitative analysis as well as the physicochemical properties of ANs.

## 2. ANs Chemistry, Biosynthesis and Stability

### 2.1. Chemistry

From a chemical perspective, ANs have a simple carbon chain (C6-C3-C6) structure, which includes them in the subgroup of flavonoids [15]. Specifically, the chemical structure composed of an aromatic ring attached to another heterocyclic ring that contains oxygen, the latter being linked by a carbon–carbon bond to a third aromatic ring is called anthocyanidin, or the aglycon of the anthocyanin [16]. In certain positions on these cyclic structures, hydrogen atoms are replaced by different functional groups [17]. When a moiety of sugar is attached to the aglycon (anthocyanidin), a new chemical compound called anthocyanin is formed [18].

**Figure 1:**
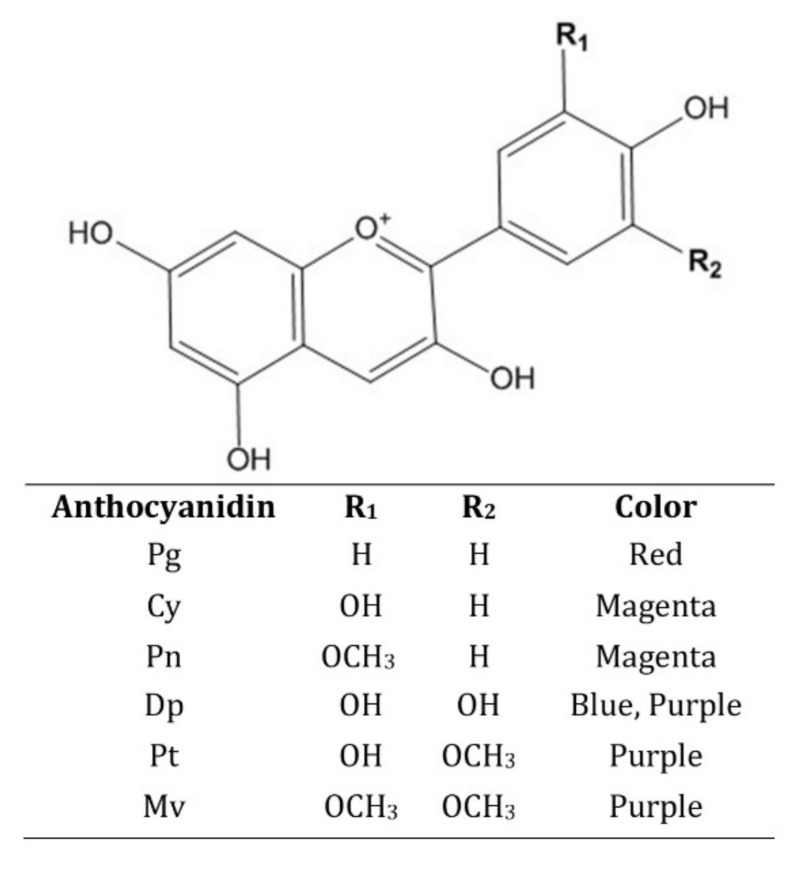
Chemical structures of the most common anthocyanidins and their color range in the visible spectrum.

Anthocyanidins are considered to be more stable than ANs. There are around 20 types of such aglycons found in nature, six of them being contained by 90% of all ANs, which are: cyanidin (Cy), delphinidin (Dp), pelargonidin (Pg), malvidin (Mv), petunidin (Pt), and peonidin (Pn) [19]. Their chemical structures and other physico-chemical properties are illustrated in Figure 1. The structural difference between these compounds is the attachment of different radicals in certain key positions on the benzene rings, which is the basis of their different physical properties. For example, Pg is responsible for color variations from orange to dark red, while Cy is responsible for colors from red to magenta and purple [20]. Pn is a compound derived from Cys, thus being responsible for colors such as purple and red-orange [21]. Dp together with its derivatives Pt (a methylated radical) and Mv (two methylated radicals) are the source pigments for the color spectrum from purple to dark blue [22].

Among these six popular anthocyanidins, Cy is the most frequent aglycon found in nature. It is regularly found as glycoside and occurs at most in sources such as chokeberries, blackberries, red cabbage, blood oranges, peaches, and plumbs [23]. Dp is abundant in grapes and, consequently, in red wine, eggplants, pomegranate, and other berries [24]. Pg dominates in beans, strawberries, radish and pomegranate, and flowers such as petunia and roses [25]. Pn was identified in cherries, blueberries, cranberries, mango, or sweet potato [26]. Mv and Pt, the least frequent anthocyanidins, are of no less importance, as they are present in important quantities in red berries, black currant, bilberries, grapes, and red wine [27].

Anthocyanidins can be further modified in versatile ways, an example being their glycosylation by attaching 1 to 3 glycosidic groups such as arabinose, glucose, xylose, galactose, and rhamnose [10]. In turn, these glycosyl moieties can be further glycosylated or acylated with various aliphatic or aromatic chemical groups [28].

The chemical nature of ANs makes them more vulnerable to degradation and structural re-arrangements, which also impact the displayed hue, due to the exposure to various environmental factors such as pH, light, oxygen, enzymes, copigmentation, chemical structure, and temperature. In these conditions, in aqueous solution, ANs can take four different forms: flavylium cation (red), carbinol base (colorless), chalcone (yellow), and anionic quinonoidal base (blue) [29]. The hydroxyl and methoxy groups of anthocyanidins act as auxochromes, electron-donating groups, which cause a bathochromic effect that results in the change of color from red-orange to purple-blue [30].

### 2.2. Biosynthesis, Genetic Regulation and Stability

For many fruits, the occurrence of ANs is a ripening indicator, since the specific color indicates the maturity of the fruit [31]. Depending on the site of production, three groups of fruits have been categorized: fruits that display ANs in their skin, fruits that store ANs both in their skin and flash, and, finally, fruits that produce ANs after an external stimulus, such as light exposure [22]. Regardless of the source, the production of specific ANs varieties is determined by the genetic background, also known as developmental regulation, while their concentration is prone to be influenced by external factors, or environmental regulation [32].

**Figure 2:**
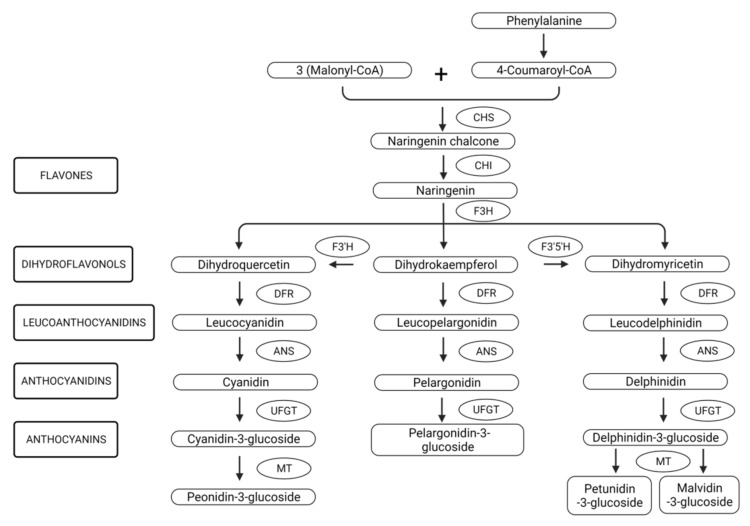
The biosynthesis pathway of ANs in plant cells. CHS: chalcone synthase, CHI: chalcone isomerase, F3H: flavanone 3-hydroxylase, F3′H: flavonoid 3′-hydroxylase, F3′5′H: flavonoid 3′,5′-hydroxylase, DFR: dihydroflavonol 4-reductase, ANS: anthocyanidin synthase, UFGT: flavonoid 3-O-glucosyltransferase, MT: O-methyl transferase (original contribution).

The synthesis of these colored pigments takes place in the cytosolic part of the smooth endoplasmic reticulum [33]. The first step in anthocyanin biosynthesis (Figure 2) is a condensation reaction between three molecules of malonyl-CoA and one molecule of 4-coumaroyl-CoA, the amino acid phenylalanine being the precursor of the last compound [21]. The reaction is catalyzed by the enzyme chalcone synthase (CHS) and the resulting product is naringenin chalcone. This reaction product is further isomerized under the action of chalcone isomerase (CHI) in naringenin [34].

Furthermore, this compound is subjected to a series of enzymatic changes under the action of hydroxylases and reductases with the formation of leuco-ANs. The production of ANs is genetically controlled by a complicated signaling pathway called the MYB–bHLH–WD40 (MBW) complex [22]. The first enzymes involved in the biosynthesis of ANs are regulated by the expression of the early biosynthesis genes (EBGs), namely: CHS, CHI, F3H, F3′H, and FLS). This action is controlled by the activity of the seven R2R3-MYB subgroups of transcription factors (MYB11, MYB12, MYB111), along with MYB75/PAP1 [35]. Furthermore, anthocyanidin synthase (ANS) contributes to the formation of anthocyanidins. Being unstable, various sugars are attached to them via methyltransferases and glucosyltransferases, thus forming anthocyanin molecules [36].

**Figure 3:**
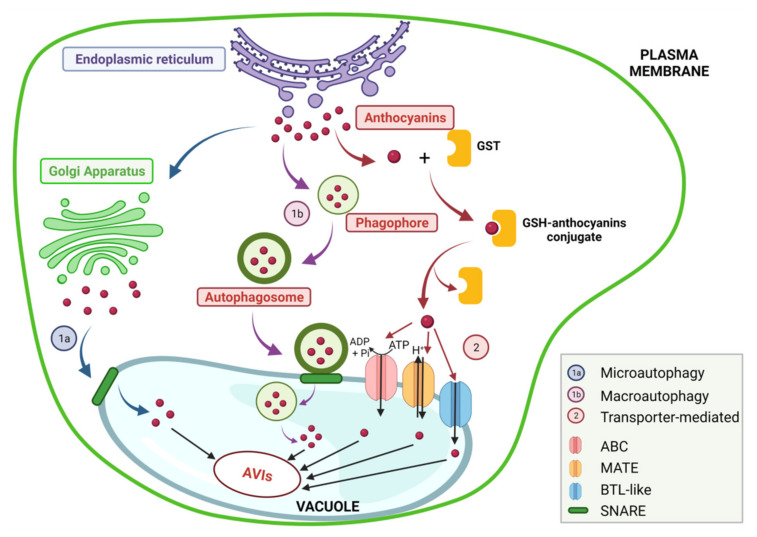
Illustration of the transportation routes of ANs post-biosynthesis, from the endoplasmic reticulum (ER) to the vacuoles. ANs travel from their synthesis location to the vacuole, for storage. The first two ways, micro- (1a) and macro-autophagy (1b), involve ER-derived vesicles, which facilitate the transit of anthocyanins to the tonoplast where they attach to the membrane through soluble *N*-ethylmaleimide-sensitive factor attachment protein receptors (SNARE) and release the ANs inside. The other transportation method is membrane transporter-mediated pathway (2) which involves several membrane proteins (MATE, ABC, and BTL-like transporters) that facilitate the transportation of ANs inside the vacuoles and their sequestration in vacuolar inclusions (AVIs). In this case, ANs are not transported in vesicles but are conjugated by GSTs (glutathione *S*-transferases) and form together the GSH (glutathione)—ANs conjugate, a stable and efficient form of transportation from ER to the tonoplast (original contribution).

Finally, ANs are transferred and stored in vacuoles by two types of mechanisms (Figure 3): vesicle-mediated transport (micro- or macro-autophagy) or membrane transporter-mediated transport (multidrug and toxic compound extrusion (MATE) transporter, ATP-binding cassette (ABC) transporter, or bilitranslocase (BTL)-like transporters) [37]. 

The enzymes involved in the final part of the synthesis, also known as late flavonoid biosynthesis genes (LBGs) such as DRF, ANS, and UFGT, as well as the membrane tonopast transporters, ABC and MATE, are activated by the upregulation of the ternary complex MBW (R2R3-MYB, bHLH, and WD40) [38]. The MBW family of transcription factors is considered to be crucial in the biosynthesis of ANs and has been identified in ripened fruits such as woodland strawberries, in large quantities, especially the R2R3-MYB protein [39]. MYB genes that belong to the MBW have also been associated with proanthocyanins production in strawberries, as FvMYB3, FvMYB9, FvMYB11, FvMYB22, FvMYB64, and FvMYB105 genes were expressed especially in the green stage of the strawberries development, which highlights the involvement of the MBW complex in different stages of ripening of the fruits [39]. Moreover, the MBW complex was also observed to participate in the activation of the EBGs in some cases such as corn (*Zea mays*) [40].

The first motif, R2R3, binds the promoter to the gene, and can both activate or inhibit the expression of a specific gene [41]. The second transcription factor, bHLH, also showed regulatory effects on gene expression and importance in ANs accumulation [42], while the third one, WD40, proved to stabilize the MBW complex [43].

The well-described molecular mechanism of ANs biosynthesis was also associated with the effects of external factors such as light exposure on ANs concentration [32]. For example, UV direct light proved an upregulation of ANs production in apples (cultivars ‘Iwai’, ‘Sansa’, ‘Tsugaru’, ‘Homei-Tsugaru’, and ‘Akane’) and strawberries (*Fragaria × ananassa* Duchesne var. Elsanta) and a more rapid darkening of the skin color [44,45]. In another study conducted on red apples (*Malus domestica*), it was observed that the *MdMYB1* gene, responsible for the coloration of the apples’ skin, was successfully transcripted when the fruit was exposed to light. However, in the dark, the protein was degraded via the ubiquitin-proteasome pathway, with a direct impact on the color of the apples, which highlights how the light controls the ANs accumulation [46].

Although the genetic regulation mechanism was well described, there are still open questions that require a deeper study. The correlation between the role of the MBW complex transcription factors and their behavior in different environmental conditions and in a wider variety of ANs sources could bring a more specific insight into understanding the developmental and environmental regulations of the biosynthesis process and their intercorrelation [22].

Considering that ANs are extremely unstable and prone to degradation, their stability is another topic of great interest to scientists, especially due to the wide range of applications demonstrated over time for them. Usually, anthocyanins are degraded due to the joint action of internal and external factors [47]. The main factors that influence their stability are: pH, light, oxygen, enzymes, copigmentation, chemical structure, and temperature [48].

#### 2.2.1. Influence of pH

ANs mainly give their source different hues from the color range of glossy orange, red, pink, purple, and blue, as determined genetically [32]. However, there are factors that greatly influence color expression, such as the pH of the environment [7]. The first aspect to consider is that ANs are reactive molecules due to the lack of electrons in the flavylium cation nucleus [20]. Due to their ionic structure, ANs are very sensitive to the pH of the environment. Therefore, at pH 1, the flavylium cation form is predominant and responsible for its red color, and, at the same time, offers the greatest stability of ANs in water [7]. At pH values between 2 and 4, the blue quinonoidal base is predominant, and as the pH increases, the colorless carbinol pseudobase and the colorless chalcone appear [18].

It is worth mentioning that at pH above 7, the degradation of the compound takes place, most ANs being more stable in acidic conditions than in alkaline ones [7]. For example, ANs from Cabernet Sauvignon red wine were investigated in simulated gastrointestinal digestion [49]. The majority of the 22 ANs initially detected in the wine were observed to be stable at acidic pH, during the gastric digestion process. However, the intestinal digestion simulation, which occurs at basic pH values, leads to a dramatic reduction in the ANs content [49]. Another study tested ANs extracted from blueberries (Britewell rabbiteye) that were exposed to 40 °C, in the dark, but at different pH values: 3.0, 4.0, 5.0, 6.0, 7.0, 8.0, and 9.0 [50]. The highest TAC (total anthocyanins content) was measured at pH 3.0 (329.459 ± 1.235 mg/100 g fresh fruit), and a continuous decrement of concentration was observed inversely proportional to the pH value: 110.388 ± 0.409 mg/100 g fruits at pH 7.0, and 49.767 ± 1.079 mg/100 g fruits at pH 9.0 [50].

The color of ANs is affected by the presence or absence of water [51]. When the water content decreases, the hydration process is reduced, being favorable for color stability. As water concentration decreases, few free water molecules are available for proton accommodation; therefore, the flavylium cation is stable [52]. In water, the pKa constant value is 3.8, while in a water-deprived environment, it decreases to 4.8, which gives a red color [53].

**Figure 4:**
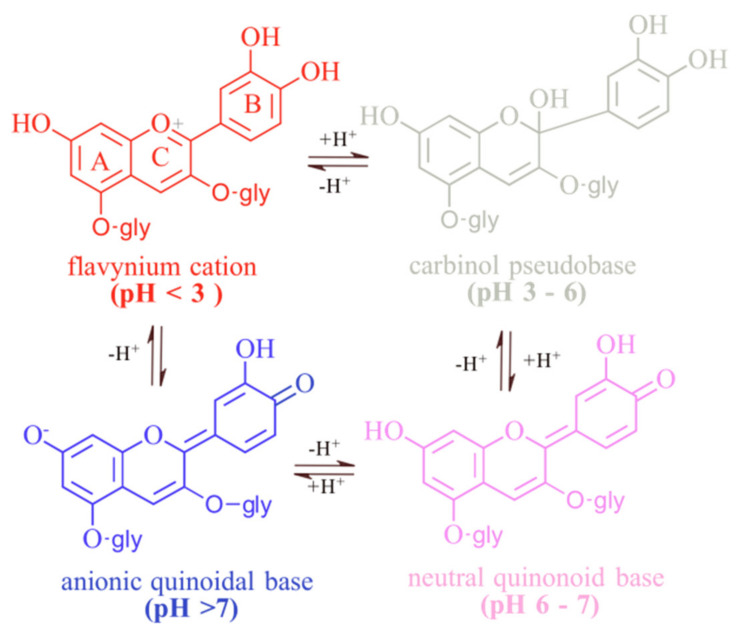
Chemical and color modifications of ANs in different pH value environments. The most representative and common form of ANs, the red-colored flavylium cation, is present at acidic pH.

As the pH value increases from slightly acidic to neutral, deprotonation of the flavylium cation starts and generates the blue quinonoidal species, highly unstable at low pH. At basic pH, the deprotonation reaction carries on and forms purple quinonoid anions (Figure 4) [54].

#### 2.2.2. Temperature and Oxygen Influence

Temperature is a key factor for better stability and preservation. The temperature at which the ANs are exposed to has great importance in the food industry, because the storage conditions of anthocyanin-rich beverages influence the quality of the product and, additionally, the maintenance or loss of ANs [55] and certain unfavorable conditions can hinder the ANs’ biosynthesis [56]. The main events that occur upon ANs as a result of thermal exposure are deglycosylation, cleavage, water nucleophilic attack, and polymerization [57,58].

A study compared the effect of two storage temperatures: 35 °C and 25 °C, and revealed that the higher temperature reduced the total content of ANs to less than half the content in grapes (*Vitis vinifera* L. cv. Cabernet Sauvignon) stored at 25 °C [59]. At 40 °C, their color changed from red to orange, despite keeping a constant acidic environment [60]. Heating the ANs shifts the equilibrium structure towards the colorless forms resulting in the loss of the desired red pigmentation.

Marquez and coworkers [38] tested three different temperature options while extracting ANs through controlled dehydration of Tempranillo red grapes: 30 °C, 40 °C, and at alternations between 40 °C and 15 °C, every 12 h. The best results were obtained in the second experiment; the grapes dried at 40 °C had the most intense color, which indicates the highest concentration of ANs. The must obtained at 30 °C still contained significant amounts of ANs but displayed a slight brown color. The least satisfactory result was obtained in the final experiment because the reduction in the temperature value allowed oxygen to diffuse through the grape skin and caused degradation reactions of ANs and loss of the red color [61].

Grape juices of ”Merlot” and ”Ruby” grape cultivars were passed through a series of experiments to evaluate ANs exposure at 2, 25, and 35 °C storage temperatures [62]. After 365 days, the ANs (Mv-3-glucoside, Dp-3-glucoside, Pt-3-glucoside, Pn-3-glucoside, and Cy-3-glucoside) were stored at 25 and 35 °C and degraded, in both cultivars (95–99%), compared to the juice kept at 5 °C (50–60%—“Merlot”; 74–81%—“Ruby”). Cy and Dp aglycones proved to have the highest sensitivity to high temperatures. There was also a strong correlation between high-temperature degradation and the change of color, highlighting that low temperatures are favorable for a longer preservation of ANs.

Moreover, exposure of Cy-3-glucoside and Cy-3-rutinoside from black rice aqueous extract to increasing temperatures from 100 to 165 °C showed a greater negative impact on ANs degradation, compared to increasing the pH value from 2.2 to 6. In addition, Cy-3-rutinoside has been proven to be the most resistant to thermal and pH changes [63].

It was also demonstrated that the degradation process of ANs is amplified by high temperatures with the presence of oxygen [62,64]. Both peel and flesh of *Malus profusion* fruits were exposed to two temperature regiments, each one divided into two intervals with an exposure time of 12 h:12 h, as follows: (1) room, RT (18 °C:25 °C), and (2) high, HT (33°C:25 °C), both in hypoxic (2%) and normoxic (21%) environments. A methanol:formic acid (70%:2%) ANs extract was obtained from frozen fruits and cy-3-galactoside was measured as the highest anthocyanin compound (≈800 µmol Kg^−1^ FW). After 1 week of treatment, RT treatments marked an increase in the cy-3-galactoside concentration as a consequence of ANs biosynthesis, regardless of oxygen conditions. However, in HT conditions, a significant concentration decrease was marked for cy-3-galactoside, with higher degradation in hypoxic conditions (67%) than in normoxic (54%).

Furthermore, HT was directly correlated with an upregulation of the *MpPOD1*, *MpPOD8*, and *MpPOD9* genes transcription, furtherly coupled with enhanced activities of peroxidases and H_2_O_2_, next responsible for the enzymatic degradation of ANs. On the other hand, maintaining samples at RT stimulated the ANs synthesis possibly by stabilizing physiological conditions such as pH and H_2_O_2_ concentration, which indicates, once more, the important role that temperature plays in the fate of ANs [65].

The presence of oxygen also brings a high contribution to ANs degradation, and it usually accompanies other factors for a more dramatic infliction on ANs stability but also works on its own. To prove this point, fresh-cut strawberries were stored in three different conditions with a focus on bioactive composition modifications [64]. Five packaging conditions were evaluated (2.5 kPa O_2_ + 7 kPa CO_2_, 10 kPa O_2_ + 5 kPa CO_2_, 21 kPa O_2_, 60 kPa O_2_, 80 kPa O_2_) for storage efficiency, for 21 days, at 4 °C. The ANs were extracted with methanol, and it was observed that their content had different behaviors, with the best antioxidant preservation at low oxygen values (2.5 kPa O_2_ + 7 kPa CO_2_). However, oxygen concentrations have mostly been linked to a postponed degradation of thermally handled ANs; namely, in the abovementioned study [64], high temperatures coupled with low oxygen concentrations led to the upregulation of POD genes, which influence other metabolic pathways that inhibit the biosynthesis of ANs [66].

Oxygen was also associated with fluorescent light as an enhancer for ANs decay [67]. Cranberry ANs were extracted with acidified ethanol and exposed for 14 days to fluorescent light, in a low oxygen environment. In oxygen-free conditions, light proved to have an insignificant effect on ANs degradation. However, when traces of oxygen were present, the pigments were oxidized and their degradation was accelerated [67]. This strongly indicates the altogether connection between different factors which act synergistically on the fate of ANs.

#### 2.2.3. Light

It is generally accepted that the production of ANs is stimulated under stress conditions when exposed to light, as was shown in several studies in the literature [68,69,70]. Moreover, ANs have the capacity to absorb light, which enables them to act as protective shields for cells such as retinal pigment epithelium (RPE) cells, against light damage, in vitro. When RPE cells were treated with different concentrations of the methanolic, purified blueberry methanolic extract (0.1, 1, and 10 µg/mL extract) and then exposed to white light irradiation of 2500 lux (420–800 nm) for 12 h, the expression of VEGF was downregulated to normal values and cellular senescence and apoptosis were suppressed [71]. On the other hand, excess light can cause photooxidation of pigments.

One such example was investigated on the fate of an ethanolic blackberry anthocyanin-rich extract with an initial concentration of 106 mg·L^−1^ Cy-3-glucoside, stored in darkness and also exposed to light [72]. One week of light exposure at 3968.30 lux determined the ANs to change color, and also induced the degradation of 76% of the total monomeric ANs concentration, while darkness storage maintained their chemical and color stability for a longer period of time and also recorded only 29% degradation of monomeric ANs [72].

However, to end on a more positive note, it was demonstrated that different light affects ANs unevenly. In one study, three different light ranges were investigated (white, red, and blue) in vivo, on strawberry fruits, in order to assess their role in ANs accumulation [73]. Strawberries were exposed to three light-emitting diodes (LED) and the ANs content was measured on the 25th day after flowering. It was demonstrated that red and blue light contributed to the high expression of key factors involved in anthocyanins biosynthesis and, therefore, led to the increase in total ANs content (TA), Pg-3-glucoside, and Pg-3-malonylglucoside concentrations, while white light treated strawberries reported the lowest concentrations of ANs [73].

#### 2.2.4. Copigmentation/Glycosylation and Acylation

Copigmentation is a phenomenon that exclusively occurs in the class of ANs among polyphenols, and contributes to their chemical stability [74]. Copigmentation is simply described as a noncovalent hydrophobic π–π interaction between an anthocyanin and a colorless organic molecule, which enhances the color and increases the stability of the molecule. For example, Dp and Pt have free hydroxyl groups on their B ring, which are easily polarizable and great sites for complexing such molecules [75].

The colorless molecules associate with the pyrylium ring of the anthocyanin without any chemical bonding. This proximity creates changes in the distribution and density of electrons, which cause UV–VIS absorption modifications of ANs [76]. These reactions can be identified as elevated λ_max_ maximal absorption wavelengths compared to the uncomplexed ANs, known as the hyperchromic effect, and also, as changes in the λ_max_ shift towards higher values (red-bluish color as bathochromic shift) which means that the complexed molecule will have a longer wavelength absorption [77]. For example, the interaction of one specific anthocyanin with different types of copigments can lead to different colorations of the molecule [75].

Three types of copigmentation have been classified so far: self-association (hydrophobic interactions between different ANs), intramolecular (association between the aromatic anthocyanidin and one or more acyl moieties, that are part of that anthocyanin), and intermolecular (Ans-colorless compounds: flavonoids, phenolic acids, amino acids, organic acids, alkaloids, purines, or Ans-metals: Al, Fe, Cu, Mg) [18,78,79]. Self-association and intramolecular reactions usually occur in flowers, whereas intermolecular copigmentation is mostly present among berries and other fruits [77].

In nature, especially in flowers, copigmentation has been optimized and engineered for optimal coloring and lasting stability. Copigmentation is also of great importance in the food industry since the association between food products and color is so strong [80]. One such example is wine, with around 50% of its color accountable for copigmentation reactions. However, in different storage conditions, a decrease in the concentration of ANs has been observed, which suggests the codependency of copigmentation with other environmental factors, such as pH or temperature [81]. ANs and copigments (total flavonols) contents were measured in Merlot and Syrah wines obtained from Greek grape cultivars, and it was discovered that after one month of storage, the concentration of anthocyanins decreased rapidly, whereas the copigments remained constant, due to their higher stability [82].

In another study, Cy-3-glucoside was copigmented with three phenolic compounds (ferulic acid, dopamine, and (+)-catechin), and their stability was evaluated according to the pigment:copigment molar ratio (1:1, 1:10, 1:100), in a pH range (pH 3–7) and at different temperatures (20, 30, 40, and 50 °C) [83]. The highest hyperchromic shift was observed in Cy-3-glucoside association with (+)-catechin, at molar ratio 1:100, pH 3 and 20 °C, followed by ferulic acid and finally, dopamine. (+)-catechin was also used to complex ANs from mulberries juice and significantly reduced their degradation during pasteurization, at 80 °C, for 15 min. The study showed that copigmentation plays an important role in maintaining the stability of ANs. However, the stability of the copigmented complex is itself dependent on various factors, among which the chemical structure of the copigment ((+)-catechin had the highest number of free hydroxyl groups), and also the temperature and the pH value of the environment, with best stability results at low values of both factors [83].

As already mentioned in the section above, the chemical structure of ANs is linked with their activity including intramolecular copigmentation. Specifically, the number, type, and position of the sugar involved in the glycosylation reaction, as well as the decoration of ANs with different types of glycosyl and acyl units, significantly influence the stability, bioavailability, antioxidant, and anticancer activity of ANs [19].

As mentioned above, anthocyanidins (the aglycones) are rarely found naturally, as they are very unstable. ANs’ chemical and physical properties are influenced and modified by glycosylation and acylation, and they change the molecular size and polarity. Water solubility is increased by glycosylation and decreased by acylation. Moreover, glycosylation increases stability due to its role in the formation of an intramolecular H-bonding network [78].

Furthermore, acylation of ANs can assure a higher stability and this form of intramolecular pigmentation structure offers protection against nucleophilic water attack [79]. Moreover, the number of acyl groups is also relevant. When compared, mono-acylated ANs from red potato showed lower stability than di-acylated ANs from red radish. A higher number of acyl groups have a “sandwich-like” structure that protects the positively charged pyrylium nucleus by positioning themselves on either side of the chromophore, due to hydrophobic interaction [74,79]. Another study that compared extracts of chokeberries, containing monoglycosylated ANs, and black carrot extracts, with 60%:40% acylated:glycosylated ANs, showed that acylated ANs had higher stability, while glycosylated showed the highest antioxidant content when extracted in methanol, possibly because the addition of acyl groups on the sugar blocks the hydrogen transfer from hydroxyl groups to the unpaired electrons. The chemical structure was also correlated with the extracting solvent since glycosylated ANs preferred methanol, whereas acylated ANs were extracted better in ethanol [84].

### 2.3. Effects of Extraction Methods on ANs

Extraction has a long history of use in the medicine and food industries and dates back to ancient times. Moreover, natural colorants sales have grown in the past 13 years at an annual rate of over 7%, in a more and more urgent attempt to replace synthetic dyes [85]. Alongside the advance in industry and technology, the market demand for a higher production rate of natural bioactive compounds implies the use of more efficient technologies to obtain higher yields of ANs [86].

The extraction of ANs is a fundamental process that refers to the conversion of a natural or raw matrix rich in ANs to a sample exclusively containing ANs [87]. An efficient extraction method for ANs includes high recovery, reduced potential of degradation or alteration, economical affordability, time-consuming optimization, and environmental friendliness [86]. However, the sensitive physico-chemical profile of ANs makes them highly susceptible to degradation and loss of stability and biological activity. In order to overcome these obstacles, vast knowledge about the factors that influence ANs’ stability and function is required [88]. Therefore, great attention should be paid to factors such as the matrix source of ANs (solid/liquid/powder), the collection time of the vegetable or its parts, the solvent of extraction, the size of the sample, the chemical structures of anthocyanins that are expected (glycosylated/acylated), the temperature and the type of extraction process, in order to provide a maximum presence of anthocyanins compounds [86,89].

By far, the most commonly used method is organic solvent extraction. The selection of the extracting solvent is one of the most important steps in achieving a proper extraction and high yield of recovery. Although methanol is the most popular choice with high extraction yield [84], it should be considered toxic and is not recommended for food or nutraceutical purposes. Searching food-friendly solvents with similar or close efficiency, it has been discovered that ethanol also assures high ANs recovery, closely followed by acetone. Water, although the most adequate for the food industry, has a very poor output in the final ANs recovery [90]. Liquid/liquid partition has also been used as a way of ANs purification, using a combination of solvents at different ratios such as acetone:chloroform/ethyl acetate/water [91]. Another crucial aspect is the acidification of the extracting solvent, in order to obtain superior recovery yield and ensure a better preservation of ANs [92]. Hydrochloric, formic, acetic, and trifluoroacetic acids are amongst the most utilized acids [85,93].

ANs are very responsive to high temperatures which affect the structure, stability, color, and composition. For this reason, ANs should be handled at temperatures as low as possible (below 40 °C) [50]. For example, in the process of solvent removal by rotavapor, most studies used 35–40 °C as the optimal temperature range for good extraction and maintenance of ANs stability, depending on the extraction method [89]. For methods that require shorter extracting time, higher temperatures could be used (100 °C), for a short time, e.g., 5 min [94].

To choose the best extraction method, a range of technologies and methods that have been described and optimized recently, sometimes difficult to make the best selection. Numerous extraction techniques approach modern and advanced technology since they represent the founding principle in the current urge to develop more rapid and efficient methods [86]. Some of the most utilized traditional extracting methods comprise maceration, digestion, infusion, percolation, reflux extraction, and Soxhlet [95]. They have been successfully used for decades, being simple without expensive materials or equipment.

Although the conventional techniques are easy to perform and are satisfactory in results, there are some downfalls such as the use of large volumes of toxic solvents for prolonged extracting amounts of time, which demand more efficient and ergonomic methods. Modern techniques have been developed with the means of replacing the tedious traditional methods that cannot ensure a scalable route for mass production [96]. Some of the most utilized modern techniques in the extraction of ANs are supercritical fluid extraction (SFE), pressurized liquid extraction (PLE), microwave-assisted extraction (MAE), ultrasound-assisted extraction (UAE), ultrasound/microwave-assisted extraction (UMAE), pulsed electric field-assisted extraction (PEF), enzyme-assisted extraction (EAE), and moderate electric field (MEF), high hydrostatic pressure extraction (HHPE), ohmic heating, and accelerated solvent extraction (ASE) [92,94,97].

## 3. ANs Bioavailability

The efficiency of ANs is strictly connected to their bioavailability [29] and specific site of action, after their ingestion and absorption [98]. In this perspective, it is noteworthy that in order to understand the link between ANs and their health-promoting actions, it is of great importance not only to know the amount of ANs from a source/treatment but also the concentration that remains bioactive in the organism.

ANs have the capacity to re-arrange their chemical structure in response to the pH of the environment. However, once they are ingested, their trail encounters several pH jumps, such as 6.7 in saliva, 1–5 in the stomach, 7.4 in the blood, and 7.5–8 in the small intestine [99,100]. Their bioavailability is thus affected, which vexes researchers as to whether their biological properties are the effect of ANs or are due to their degradation metabolites.

In an approach to overcome this underexplored subject, several studies have been conducted on the fate of ANs after ingestion, both dietary (from natural sources) [6] and as supplements [6,101,102]. Their findings revealed that ANs could be identified in native (glycosylated) forms in the plasma, in the stomach and could also be stored in some tissues and organs such as the liver and eyes, and even pass the blood–brain barrier to the cortex and the cerebellum, in pig subjects [103]. In several studies performed in humans using a variety of anthocyanin-rich sources, the concentration of plasma compounds in the range of 6–2400 mg has been reported [97,104,105,106,107]. Similarly, an in vivo study in rats showed the presence of ANs in the stomach at a concentration of 2013.2 ± 280.2 μM at 0.5 h after administration of a dose of 500 mg/kg dose of Cy-3-glucoside [108]. Moreover, the absorption of 23.0 nmol of ANs was recorded 30 min after the injection of a concentration of 70.9 nmol in the gastric lumen of rats [101].

Further, ANs metabolites were observed in other organs such as the liver, heart, jejunum, kidneys, bladder, prostate, testes, and fat tissue, in rats [102]. The rapid breakdown was also observed in human subjects with 56% of the metabolites of Cy-3-glucoside still present in the circulatory system after 48 h from ingestion. The maximum concentration obtained, measured in μmol/L, was: 5.21 ± 3.43 for hippuric acid, 1.51 ± 0.75 for phenylpropenoic and phenylacetic acids, 0.48 ± 0.10 for protocatechuic acid and phloroglucinaldehyde, 1.17 ± 0.52 for phase II conjugates of Cy-3-glucoside and Cy, and 5.54 ± 0.49 for phase II conjugates of protocatechuic acid [109].

It has been thus observed that ANs are absorbed in different manners according to their molecular size, aglycone, and the glycosylation and/or acylation feature, as well as the pH of the environment. Moreover, it has been discussed that the absorption is also influenced by the concentration of ANs administered, this was pointed out in a study of 13 healthy volunteers who consumed 0.8 mg of ANs/kg of body weight from chokeberry juice [110].

**Figure 5:**
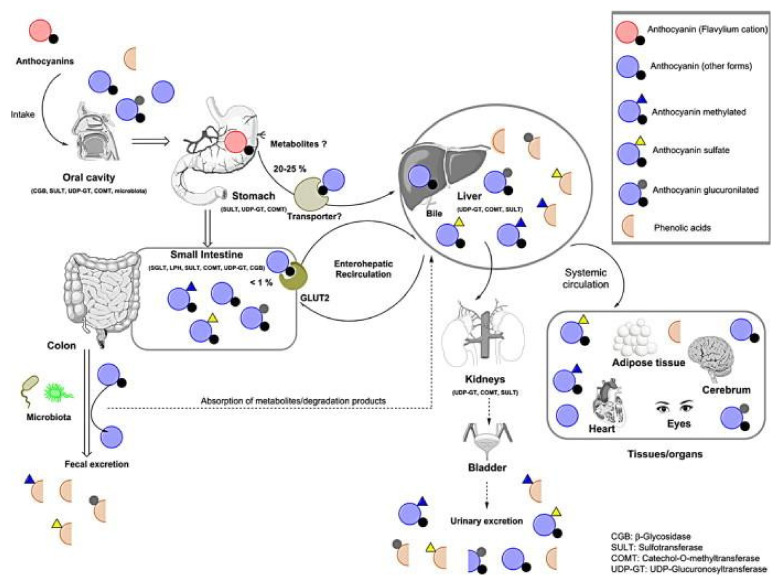
Proposed scheme for the metabolism, distribution, absorption, and excretion of ANs. Copyrights Fernandes et al. [99].

Once ingested, ANs follow the continuous path of the gastrointestinal tract of different physico-chemical settings (saliva, stomach, intestine, and colon), and different populations of microorganisms. Following the absorption, they enter further compartments and environments (Figure 5), which all put a toll on the stable flavylium cation and, consequently, on the final concentration of ANs that remains available in the organism [111].

### 3.1. Oral Cavity Absorption

The interaction of ANs with digestive enzymes or food proteins is the first transformation they undergo in the oral cavity. A study by Walle et al., 2005, demonstrated the hydrolysis of flavonoid glucosides to aglycones under the action of β-glucosidases derived from bacteria and oral epithelial cells of human subjects [112]. In another study, ANs extracted from black peanuts skins exerted an inhibitory effect on the digestive enzymes with IC50 values of 123.4 μg mL^−1^ for α-amylase and 82.75 μg mL^−1^ for α-glucosidase [113]. The interaction between different proteins and ANs has been studied in several studies. For example, Mv-3-galactoside in blueberry extract was stabilized by binding to α-casein or β-casein. In turn, these proteins have undergone structural changes in α-helix, β-sheet, turn, and random coil content [114].

Several studies have shown a lower rate of absorption in the body when compared to the amount administered orally, due to the very low percentage measured in plasma, shortly after ingestion [99,115]. Generally, 1% of the total content could be found in the circulatory system, after 15–30 min post-ingestion [116]. However, parts of ANs can be subjected to degradation to metabolites, or could bind to plasma proteins, or could be uptaken by cells from mouth tissue [99].

Although the intestinal lumen and the liver are considered to be the main absorption and digestion sites of ANs, small quantities could be recovered in the mouth too. Most of the ANs are degraded as a result of enzymatic and microbiota actions. The process was mostly dependent on the chemical structure of ANs. For example, a clinical trial showed that ANs from chokeberry juice had different fates, after 5 min of incubation in the oral cavity. Cy-3-xyloside had the lowest recovery yield, hence the highest deterioration, while Cy-3-glucoside could be identified in the epithelium cells [117]. However, there are possible oral components that contribute to the intraoral metabolism of ANs such as: saliva, oral microflora, and oral tissues [99,118]. Such effects have been observed in humans who were administered black raspberries ANs [118]. Native forms of ANs as well as degraded forms were identified in the saliva and in the oral cavity.

The mechanism behind their uptake in the mouth tissue is believed to be attributed to enzymes such as sodium-dependent glucose co-transporter (SGLT1), β-glycosidase (derived from oral bacteria and epithelial cells), UDP-glucuronosyl-transferase, catechol-O-methyltransferase (COMT), which contribute to the absorption of ANs, increasing water solubility or their hydrolyzation to simple aglycones [118].

Another study suggests that ANs degradation in the saliva is mostly mediated by the resident microbiota, a network of over 700 kinds of microorganisms, mostly composed of bacteria, fungi, and viruses [119,120].

Among the metabolites found in the saliva, several studies could identify low amounts of Cy, Dp, Pn, Mv, and mostly, phenolic compounds as a result of degradation such as protocatechuic acid or chalcones of Cy, remarking that the chemical structure of ANs and the amount of salivary secretion strongly influenced their chemical degradation and their uptake by the epithelial cells [117,119].

### 3.2. Gastric and Intestinal Absorption

#### 3.2.1. Stomach

In the stomach, the pH range is around 1–2, which favors the preservation of ANs in the form of cation flavylium, their most stable form. This hypothesis has also been confirmed by numerous in vitro studies [121,122,123]. The stomach is considered a highly effective site, and a larger amount of ANs is absorbed (20–25%) here, rapidly passing into plasma, and then excreted into bile and urine [124]. Moreover, no secondary metabolites were identified in the stomach after 30 min, only the native (glycosylated) form of ANs. In one study, rats were administered black raspberry anthocyanin extract through a gastric tube, and the flow of ANs was kinetically measured in the stomach. The concentration decreased linearly for 180 min. After 120 min, the concentration was halved. Therefore, the authors suggest that after 4 h, anthocyanin forms should still be detected, even if they are in small concentrations of approximately 10% of the ingested dose, as demonstrated in another article [125]. In addition, another aspect of measuring ANs is their binding to other stomach proteins, such as an anion membrane protein transporter, named bilitranslocase [126]. Since HPLC is able to detect free ANs, this again would lead to a misinterpretation of the real concentration of ANs in the stomach [116].

Some of the main transporters that are considered to be involved in the stomach absorption of ANs include glucose transporter 2 (GLUT2), which was observed to be increased in cells pre-treated with ANs compared to untreated cells, highlighting a circle that would improve their bioavailability by steadfast consumption [121].

Regarding the stomach, the literature gives vast and ambiguous information. An in vitro model is needed that acts as a gastric barrier and can analyze the absorption of ANs at low pH. In general, the stomach is ruled out when it comes to absorption tests, but it is a very important site of absorption [99]. The amount of ANs that is not absorbed by the stomach is delivered to the small intestine mostly as carbinol pseudobases [14].

#### 3.2.2. Intestine

The ANs that escape the stomach absorption reach the small intestine. Due to the basic conditions, they will be mainly found as colorless pseudobases, yellow chalcone, or blue quinoidal forms [14]. Moreover, they are rapidly subjected to degradation into secondary metabolites, which are then released into the circulatory system and finally excreted in the urine. The unabsorbed ANs continue their route towards the colon, where they suffer modifications under the action of the microbiota environment, which hydrolyses the glycosidic forms of ANs into aglycones and later, into simple phenolic acids [127]. The secondary metabolite protocatechuic acid has accounted for the majority of the ANs ingested. However, some other forms of metabolites have been identified and are presented in Figure 3.

The mechanism by which ANs may be absorbed into the intestinal epithelial cells is, again, through glucose transporters such as GLUT2 [36] and possibly SGLT1 [128].

ANs, once ingested, follow a particular pathway, starting with the oral cavity where, mostly influenced by their chemical structure, they can be degraded by the activity of saliva enzymes and microbiota or can be absorbed by oral mucosal epithelium [117]. The highest amount reaches the stomach where, due to optimal pH conditions, they can be recovered in their specific flavylium cation form and be absorbed into the plasma [129]. They can also be absorbed by the small intestine, where they undergo decomposition under the action of the intestinal microflora [130]. The secondary metabolites and degradation products can have important medicinal properties and are taken up by the liver and then distributed to other organs and tissues [131].

## 4. ANs and Preventive Action on Diseases

Due to their occurrence in many fruits, ANs have been part of the human diet for years and have been used in traditional medicine to treat several impairments, such as atherosclerosis, chronic venous insufficiency, obstructed bile ducts, hyperbilirubinemia, and lack of appetite. They were also used during World War II by British pilots who were administered bilberry jam for better night sight [122,123].

These pigments have gained a new appreciation over the past decades, as the consumption of an ANs-rich diet has been associated with positive health reactions [132]. Consequently, the amount of research on this topic has intensified significantly over the last two decades, which has helped attain more and more insights into ANs’ properties and their antioxidant and anti-inflammatory actions [133]. This naturally led studies to extend to a wider range of pathologies, such as: diabetes, obesity, cardiovascular diseases, neuronal illnesses, and cancer [134]. The main mechanism by which ANs are believed to have the ability to prevent the development of diseases is related to their antioxidant capacity by which they diminish prooxidative damage [135].

Reactive oxygen species (ROS) such as hydrogen peroxide (H_2_O_2_), hydroxyl radical (HO•), and superoxide anion (O_2_^−^) are reaction products that are generated by the body as a result of a partial reduction of oxygen in mitochondria oxidative phosphorylation or as a response to drug metabolites, bacterial invasion, or infections. The excessive production of ROS, which may commence endogenously in pathological conditions, or from exogenous sources (pollution, UV-B radiation, smoking, unhealthy diet) is called oxidative stress [136]. Oxidative stress is a defective imbalance in the redox mechanism in which the cell is ineffective in mounting an antioxidant response against ROS which, in turn, generates an excessive ROS production [137].

Furtherly, an overwhelming amount of ROS affects biomolecules of critical importance such as proteins, lipids, and nucleic acids which further causes obesity, diabetes, cardiovascular associated disorders, carcinogenesis, neurodegeneration, and aging, as comprised in Figure 2 [138,139]. These ROS-mediated damages go hand in hand with inflammatory processes such as the activation of specific signaling pathways responsible for enhancing the expression of pro-inflammatory cytokines and mediators such as: interleukins (IL), C-reactive protein (CPR), tumor necrosis factor (TNF), vascular cell adhesion molecule-1 (VCAM-1), intercellular adhesion molecule-1 (ICAM-1), etc. Although beneficial in the host response to infections, these mediators, under pathological conditions, trigger signaling cascades that lead to severe illnesses such as the ones mentioned above [140,141]. By controlling the levels of expression of these pro-inflammatory effectors, the risk of the later development of chronic diseases could be reduced.

### 4.1. In Vitro Studies

Cell cultures are valuable tools that provide reliable, predictable systems that faithfully reproduce live models, and at the same time circumvent some obstacles and limits that arise in in vivo research, which enabled scientists to conduct an in-depth study of ANs (Table 1).

Therefore, a growing body of studies over the past two decades has demonstrated the actions of ANs on different pathologies [142].

The excessive accumulation of adipose tissue and obesity is the strongest risk factor for developing diabetes [170], therefore, the two chronic pathologies are positively correlated. In addition to the abnormal metabolic disturbances, there are some particular features as well [171]. In this context, ANs have been shown to be beneficial in modulating and preventing their occurrence in numerous studies.

Many different in vitro models have demonstrated the potential of ANs to alter different molecular aspects of diabetes and obesity [143,144,145]. A molecular sign of obesity is represented by pro-inflammatory macrophages that infiltrate the adipose tissue, which further leads to a high production of pro-inflammatory cytokines (interleukins IL-6, IL-8, and 1L-1β or tumor necrosis factor α (TNF-α) and chemokines such as C-C motif chemokine ligand 2 (CCL-2) [172]. To investigate the relationship between adipocytes and macrophages, anthocyanin-enriched fractions from blackberry–blueberry beverages (100 μM) were applied to two cell lines: RAW264.7 macrophages and preadipocyte 3T3-L1 cells. After 24 h of treatment, markers such as phosphorylated-p65 NF-ƙB (51.2%), nitric oxide (17.5%), and TNF-α (89.4%) were inhibited in RAW264.7 cells. Furthermore, in mature 3T3-L1 adipocytes, isoproterenol-induced lipolysis and intracellular fat accumulation were reduced by 18.6% and 28.2%, respectively [143].

In another study, hepatocellular carcinoma cell line HepG2 cells showed a significant inhibition of reactive oxygen species (ROS) production after pre-treatment with 5 μg/mL ANs (blueberry anthocyanin extract (BAE)), Mv, Mv-3-glucoside, or Mv-3-galactoside for 24 h. Decreased production of enzymes involved in lipogenesis and gluconeogenesis has also been observed, while those involved in glycogenolysis and lipolysis have been enhanced via the adenosine monophosphate-activated protein kinase (AMPK) signaling pathway [144].

The protective role of ANs on endothelial cells has been demonstrated in a study by Aboonabi et al. [145]. Specifically, after the application of 50 μL/mL of berry-derived ANs on diabetic human aortic endothelial cells (D-HAEC) subjected to oxidative stress, a decrease in the inflammatory and oxidative process was demonstrated by inhibition of the NF-ƙB signaling pathway [145]. Similarly, the anti-inflammatory and antioxidant effects of anthocyanin extracts from various fruits have been demonstrated in retinal epithelial cell models [173,174,175,176].

Improving the quality of life and the life expectancy of human beings has been a remarkable achievement of the continuous progress made by humankind in recent decades. However, managing this situation is difficult, mainly due to the significant increase in the number of people suffering from chronic diseases. According to a 2021 WHO report, 71% of all deaths globally in one year are caused by such diseases. Of the 41 million deaths a year, 17.9 million are caused by cardiovascular disease. One way to prevent these pathologies is to choose a healthy diet of overprocessed food. Although the term “healthy diet” has not been universally defined, there are guidelines that can be applied. Among them, the consumption of vegetables and fruits, especially fresh and raw, is strongly recommended by the WHO [177].

The effect of ANs on the expression of genes responsible for the inflammatory response has recently been highlighted [178]. Human umbilical vein endothelial cells (HUVECs) have been exposed to several types of ANs in concentrations from 0.1–2 μM. Subsequent analyses have shown that these bioactive compounds can reduce the adhesion of monocytes to endothelial cells, the initial step in atherosclerosis development.

Contrary, no effect has been identified on the expression of genes encoding the adhesion molecules E-selectin, ICAM1, and VCAM1, indicating that the target proteins for this process are different [146]. In a similar study, TNF-α-stimulated HUVECs treated with 100 or 300 µg ANs for 6 h demonstrated a significant inhibition of TNF-α-induced NF-κB activity with translocation of NF-κB from the nucleus to the cytosol [147]. NF-κB is a transcription factor that regulates gene expression of adhesion molecules on endothelial cells and the production of pro-inflammatory cytokines and chemokines [179].

An in vivo study focused on evaluating the effect of ANs on apolipoprotein E-deficient (apoE^−/−^) mice to investigate the early stage of atherosclerosis. Following nutrigenomic analysis, 1261 genes were identified whose expression was modulated by bilberry anthocyanin-rich extract (0.02%). Genes encoding enzymes involved in the regulation of oxidative stress (AOX1, CYP2E1, or TXNIP) and some encoding adhesion proteins (JAM-A, VEGFR2) were down-regulated, and genes responsible for cell–cell adhesion were up-regulated. (CRB3, CLDN14, CDH4) [180]. A significant upregulation of the Nrf2-regulated antioxidant response proteins heme oxygenase 1 (HO-1) and glutamate-cysteine ligase subunit modifier (GCLM) was recorded after treating HUVECs with blueberry juice in response to low µM concentrations of H_2_O_2_ (0–40 μM) [148].

The CD40-mediated signaling pathway contributes to the trigger of atherosclerosis. To examine the role of ANs on cell adhesion, HUVECs were stimulated with 5 μg/mL sCD40L and subsequently treated with Cy-3-glucosides and Pn-3-glucosides from 1 to 100 μM for 24 h. The level of VCAM-1, ICAM-1, MMP-1, and MMP-9 decreased significantly after treatment. Caspase-3 activity, involved in cellular apoptosis, and JNK and p38 pathways were also inhibited [149]. Oxidized low-density lipoprotein (oxLDL) may enhance immune and inflammatory mechanisms that promote atherosclerosis [181]. oxLDL inhibition was achieved by applying a mixture of ANs from *Hibiscus sabdariffa* L. over mouse macrophage J774A.1 cells.

Moreover, with the treatment in concentrations of 0.01–0.2 mg/mL, it was possible to decrease the CD36 expression, both at the protein level and at the mRNA level [150]. CD36 is a membrane protein with a pivotal role in modulating lipid metabolism; often, its overexpression is correlated with the accumulation of toxic lipids and a high risk of heart failure [182].

Another extremely important role that ANs have shown in various studies has been in the fight against cancer. As it is known, the treatments for this disease are not yet well established, so new therapeutic options are being sought with interest by scientists. The literature offers us various in vivo and in vitro studies, combined, that aim to test such a treatment, in different therapeutic strategies. For example, human hepatoma cell line SMMC-7721 treated with 0.2 mg/mL of ANs from *Lonicera caerulea* ‘Beilei’ fruit could inhibit the proliferation of cells and also promote their apoptosis. Furthermore, H22 tumor-bearing mice treated with the same extract, in different doses, were analyzed. Tumor growth was suppressed by activating SOD and decreasing the amount of MDA, IFN-γ, and IL-6 [151].

Grape seed extract (GSE) has been tested for chemically induced liver cancer. When administered at doses of 25, 50, and 100 mg/kg per day for 14 weeks, the extract produced effects such as reduced tumor cell proliferation, oxidative stress, and the concentration of inflammatory markers such as iNOS, cyclooxygenase 2, and p-phosphorylated tumor necrosis receptor factor in the rat animal model. In HepG2 cells, the grape seed extract facilitated the activation of caspase-3 and G2/M and G1/S cell cycle arrest [152].

The anticancer activity of Dp in colorectal cancer has also been investigated, both in vitro and in vivo. The human colon cancer cell lines DLD-1, SW480, and SW620 were incubated with serial concentrations of Dp (<100 μM) for 24 h. The main results obtained indicated the inhibition of cell adhesion, migration, and invasion of tumor cells and epithelial-to-mesenchymal transition. Integrin and FAK signaling cascade were successfully inhibited in the DLD-1 cell line treated with 100 μM Dp for 24 h. At the mRNA level, miR-204-3p is upregulated in response to Dp. An animal xenograft model was used to analyze the effect of ANs in vivo. Here, the attenuation of the metastasis process could be highlighted [153].

Similarly, the effect of Dp-3-glucoside and Cy-3-glucoside were tested on HCT-116 and HT-29 human colorectal cancer cells (100–600 μg/mL). Programmed cell death protein-1 (PD-1) and programmed death-ligand 1 (PD-L1), important immune checkpoints, have been successfully inhibited by these compounds [154]. Colon cancer stem cell proliferation was attenuated by the application of 5 μg/mL purple-fleshed potato extracts, rich in ANs. Furthermore, the phenomenon of apoptosis has been accelerated by modulating the BAX protein [155].

There are also many results that suggest the applicability of ANs in the treatment of melanoma. The antiproliferative activity of ANs-rich strawberry extracts and their potential to induce differentiation have been successfully tested in vitro on the B16-F10 cell line [156]. The antioxidant capacity of ANs has also been shown. Mulberry ANs extract applied to B16-F1 cells helped to reduce the expression level of phosphoinositide 3-kinase (PI3K), Ras protein, and NF-ƙB within 24 h [157].

In breast cancer, an anthocyanin-rich extract from black rice has shown therapeutic potential. Specifically, the viability of breast cancer cell line MCF-7 was suppressed, and, at the same time, their apoptosis was stimulated by activating caspase-3, depolarizing the mitochondrial membrane, and releasing cytochrome c. In the same manner, in an in vivo model of mice bearing MDA-MB-453 cell xenografts, inhibition of the angiogenic factors matrix metallopeptidase MMP-9, MMP-2, and uPA was performed after the oral administration of 100 mg/kg/day of ANs extract [158]. Different concentrations of ANs from red sorghum bran (250 μg/mL, 500 μg/mL, and 1000 μg/mL) were applied over the human breast cancer cell line MCF-7. Subsequently, morphological changes associated with apoptosis were identified by microscopic methods, thus demonstrating inhibition of tumor proliferation [159].

Therefore, ANs can be applied in order to alleviate various forms of cancer. Of course, it is essential that research continues in order to finally reach a product that cures this pathology.

Another beneficial effect of ANs in chronic diseases is to provide a certain degree of neuroprotection, helping in the fight against neurodegenerative diseases. These are more common among the elderly population because they are caused by genetic and environmental factors that, over time, have an increasing impact on the proper functions [183]. The most common diseases in this category are Alzheimer’s disease, Huntington’s disease, Parkinson’s disease, frontotemporal dementia, amyotrophic lateral sclerosis, and spinocerebellar ataxias [184]. Even if the exact causes that trigger the phenomenon of neurodegeneration are not known, there are several scientifically documented variants that can explain the appearance of the pathologies. One variant is based on molecular studies and indicates the accumulation, over time, of aggregates formed by the proteins amyloid-β (Aβ), hyperphosphorylated tau (p-tau), and α-synuclein [183].

The use of ANs to prevent these pathologies was reported, ANs being rapidly absorbed into the bloodstream and easily cross the blood–brain barrier (BBB), thus exerting their beneficial effects in their unaltered form (anti-apoptotic, antioxidant, and anti-inflammatory) [185].

The neuroprotective effect of Cy (0.2–20 μM) was tested on human neuroblastoma SK-N-SH cells treated with Aβ25-35, a neurotoxicity-inducing compound. After incubation with the target anthocyanin for 2 h, results such as decreased ROS accumulation, modulation of intracellular Ca^2+^ amount, decreased expression of ER stress response proteins, and transcription factors such as eukaryotic initiation factor 2 α (eIF2α), X-box binding protein 1 (XBP-1), and cleaved caspase-12 [168]. Similarly, human SH-SY5Y neuroblastoma with hydrogen peroxide-induced neurotoxicity was treated with a series of concentrations of blueberry and cranberry juices for 24 h. Subsequent tests showed an increase in the activity of the antioxidant enzymes catalase (CAT) and superoxide dismutase (SOD), complemented by a decrease in ROS and TBARS [169].

### 4.2. In Vivo/Clinical Studies

ANs studies have extended from cell cultures to a variety of in vivo models, animal and human clinical trials, as describes in Table 2.

Concerning diabetes, there is much in vivo evidence that shows how ANs protect the pancreatic β-cells, decrease glycemia, enhance insulin secretion, suppress weight gain, decrease hepatic lipogenesis or accumulation in the liver, reduce adipocytes sizes, enhance insulin resistance and sensitivity, reduce total cholesterol, and increase levels of HDL [170].

A cohort study of 16,678 healthy people from the US National Surveys Study (REasons for Geographic and Racial Differences in Stroke) found that a diet rich in ANs and proanthocyanidins helps reduce the incidence of coronary heart disease [213]. Consumption of 400 g of fresh bilberries by 15 volunteers with features of metabolic syndrome has been shown to be useful in lowering the concentration of pro-inflammatory biomarkers IL-6, IL-12, and C-reactive protein. Moreover, following a transcriptomic analysis, results were obtained confirming a significant decrease in the expression of key genes in macrophage differentiation and activation (MMD and CCR2) [186].

An 8-week placebo-controlled clinical trial tested the therapeutic potential of anthocyanin-rich black soybean test extracts (2.5 g/d), with high concentrations of ANs (12.58 mg/g), on 63 obese volunteers. Changes in plasma lipid profile have been reported, such as decreased low-density lipoprotein cholesterol (LDLc) triacylglycerols (TG), and non-high-density lipoprotein cholesterol (non-HDLc) [187]. Furthermore, 124,086 people living in the US were included in a clinical study that aimed to verify the association between the consumption of specific flavonoids (ANs, flavonols, flavones, flavanones, flavan-3-ols, and flavonoid polymers) and weight change over time (1986–2011). ANs showed the highest magnitude of association, the main food sources consumed being blueberries, strawberries, apples, and pears [188].

On the other hand, consuming 500 mL of red-orange juice for 12 weeks had no effect on the weight of the study volunteers. However, the LDL concentration decreased significantly [189]. Similarly, the administration of 118.5 mg/day of ANs from dried purple carrot to 16 obese men for 4 weeks did not show changes in body mass or molecules of interest such as LDL, total cholesterol, and C-reactive protein [214].

Going further in the field of in vivo studies conducted on this topic, it can be stated that there is a fairly large diversity of such research.

In vivo tests have been performed to further analyze the potential applications of ANs in the treatment of diabetes and its macrovascular (cardiovascular) and microvascular (retinopathy, neuropathy, kidney disease) complications [215]. For example, 200 mg kg^−1^ of raspberry anthocyanin was administered for 12 weeks in C57BL/6 mice with a low-fat diet or high-fat diet. Following analysis, a significant decrease in gene expression was observed for TNFα, IL-6, and NF-κB. At the same time, the activity of the antioxidant enzymes superoxide dismutase (SOD) and glutathione peroxidase (GSH-PX) increased significantly, and, moreover, the body weight was reduced by 63.7% [190].

ANs from black rice, black soybean, or purple corn were tested in C57BL/6 mice fed a high-fat diet. The dose administered for 12 weeks was 200 mg/kg and showed a decrease in lipid peroxidation and gene expression for TNFα, IL-6, iNOS, and NF-κB and an increase in peroxide dismutase and glutathione peroxidase activity [191]. In comparison, extracts of ANs from mulberry and cherry showed similar effects under the same experimental conditions [192].

The metabolic syndrome has also been investigated in the animal model. In obese Zucker rats who followed a diet containing 8% wild berries for 8 weeks, there was a significant decrease in the molecular markers IL-6, TNF-α, and Nf-κB in both the liver and adipose tissue and the reactive protein C in the liver. At the same time, a higher concentration of adiponectin, a well-known homeostatic factor that regulates glucose levels, has been observed [193]. The inhibitory effect of Cy-3-glucoside on retinol-binding protein 4 (RBP4) expression and the enhancing effect on glucose transporter 4 (Glut4) in the adipose tissue were highlighted in KK-A(y) type 2 diabetes mice after a diet with 0.2% anthocyanin for 5 weeks [194].

Another potential anti-diabetic role that anthocyanin-rich extracts may play is glycosidase action. To test this hypothesis, 24 adult male Wistar rats that had a cornstarch or fructose-rich control diet for 4 weeks, received an additional 2 g/kg Kamchatka honeysuckle berry extract (327 mg ANs/g). The conclusions of the experiment showed a stimulating effect on bacterial α and β glucosidase activity within the gut [195]. C57BLKS/J-Lepr^db^ mice treated with 10 mg/kg BW anthocyanin-rich purple corn extract for 8 weeks were subsequently analyzed to investigate the effects of the bioactive compound in diabetic nephropathy. Thus, the induction of VEGF and HIF-1a transcription factors that promote angiogenesis was successfully attenuated. Furthermore, the mesangial and endothelial induction of angiopoietin (Angpt) proteins under hyperglycemic conditions was successfully decreased [196].

Dp-3-rutinoside from blackcurrants extract demonstrated activating action on glucagon-like peptide-1 (GLP-1) in an in vivo study performed on Sprague-Dawley (SD) rats. GLP-1 is a gastrointestinal peptide involved in glucose homeostasis and the amount of extract administered (5 mg/kg BW) showed positive results in the secretion of the enzyme of interest [197].

Therefore, there is a lot of scientific evidence to support the use of ANs in antidiabetic therapy. However, continuous innovation is needed to cope with the growing number of patients suffering from this disease.

Several epidemiological studies have shown a negative correlation between the incidence of cardiovascular disease and the consumption of anthocyanin-rich fruits and vegetables. For example, the findings of a study looking at the relationship between anthocyanin intake and the risk of myocardial infarction (MI) in 93,600 women between the ages of 25 and 42 showed an inverse association between high consumption of this bioactive compound and the risk of MI [198]. In another study, high blueberry consumption contributed to increased flow-mediated dilation (FMD) and improved vascular function in 21 healthy men [199].

A significant decrease in cholesterol, triglycerides, and activated platelets and an increase in total plasma antioxidant capacity were observed after 30 days of consumption of strawberries by healthy volunteers, in a clinical study [200]. In a clinical trial study, 150 subjects with hypercholesterolemia consumed a purified anthocyanin mixture (320 mg/d) or a placebo twice a day for 24 weeks. After this period, the levels of serum high sensitivity C-reactive protein (hsCRP), soluble vascular cell adhesion molecule-1 (sVCAM-1), and IL-1β were measured. The results indicated a significant decrease in the amount of these biomarkers in plasma [201].

In the category of epidemiological studies, we find a study that showed the pro-apoptotic effect of an anthocyanin-rich dietary bilberry extract in 30 patients with chronic B cell lymphocytic leukemia and on peripheral blood mononuclear cells (PBMCs) from healthy subjects. The main compound in the extract, Dp-3-glucoside, causes the activation of caspase-3 and the down-regulation of the Bcl-2/Bad pathway [202]. In vivo studies have shown the potential of Dp to significantly reduce melanoma-induced tumor growth, while in vitro data show a decrease in endothelial cell proliferation [203]. Protective effects of Cy-3-glucoside and Dp against UVB irradiation have been reported in in vivo studies on the carcinogenesis model of hairless mice SKH-1 [204,205].

#### Neuroprotection Sustained by ANs

In the field of clinical trials, a study was conducted on 49 older adults (+70 years with mild-to-moderate dementia who consumed for 12 weeks 200 mL/day of anthocyanin-rich cherry juice. Compared to the control group, those with dementia improved their cognition, respectively, fluency in speech, and short- and long-term memory. On the other hand, inflammatory molecular markers did not undergo significant changes [206].

Another randomized, double-blind, placebo-controlled trial looked at effective freeze-dried blueberry consumption in elderly volunteers. According to the researchers, the addition of anthocyanin compounds to the diet helped to reduce verbal errors and significantly reduced switch costs on a task-switching test [207]. Twenty-one children aged 7–10 were volunteers in an experiment that aimed to verify the effects of the introduction of 15 or 30 g of freeze-dried wild blueberry powder in the diet, demonstrating cognitive improvements [208].

ANs extracted from Korean black soybean (100 mg/kg ANs) were applied for 7 weeks to an in vivo model using Sprague-Dawley (12 weeks old) male rats. Surprisingly, memory deficits were improved; the expression of RAGE, BACE-1, and Aβ proteins was inhibited, as was the amount of MDA. Furthermore, the activation of astrocytes and microglia in the brains of the tested rats was observed [209]. Male C57BL/6N mice underwent intraperitoneal injections of LPS (250 μg/kg/day for 1 week) to simulate neurotoxicity and neuroinflammation. Subsequently, they received anthocyanin treatment extracted from the same Korean black soybean (24 mg/kg/day) for 14 days. Inflammatory markers p-NF-κB, TNF-α, and IL-1β were down-regulated and cellular apoptosis was reduced by suppression of inducing factors (cytochrome C, cleaved caspase-3, and Bax) [210].

Strong activation of antioxidant enzyme activity in the cerebral cortex was demonstrated by the administration of ANs (200 mg/kg/day) in an experimental model of sporadic dementia of Alzheimer’s type that used adult rats [211]. In another study, after administering chokeberry ANs for 15 or 30 mg/kg chokeberry ANs to Kunming mice receiving D-galactose to support their aging for 8 weeks, the redox balance was restored, DNA fragmented and the amount of pro-inflammatory cytokines COX2, TGF-β1, and IL-1 decreased [212].

Therefore, the protective role of ANs in the fight against degenerative diseases may be of great interest for future research, especially due to the multitude of scientific articles that come with convincing evidence in this regard.

### 4.3. Medicinal Products Developed with ANs

ANs have a very high versatility in terms of their applications and their beneficial effects on human health. Therefore, in addition to using them in free or encapsulated form, there are also topical formulations that have been successfully created. For example, a combination of ANs extracted from *Zea mays* and *Clitoria ternatea* was incorporated into a mucoadhesive gel. This product was subsequently tested and evaluated for topical oral wound healing in rats and in a clinical trial in 68 orthodontic patients. In the animal model, a reduction in the erythema and the sizes of oral wounds was observed compared to placebo gel. In the clinical trial, wound closure enhancement was observed on day 3 of anthocyanin gel application [216].

Another study tested the ability of pomegranate ANs to prevent aging by formulating a cream. After obtaining favorable results in terms of stability, homogeneity, and ex-vivo studies, the cosmetic product was tested on volunteers. Features such as wrinkle reduction, hydration, and pleasant texture have been demonstrated following the topical application of the cream [217].

## 5. Conclusions and Future Outlook

ANs are a class of water-soluble phytonutrients that are widely distributed in fruits and vegetables and show valuable health effects. Their chemical stability is highly susceptible to degradation and is directly dependent on environmental conditions. This particularity is especially observed when investigating the faith of ANs after ingestion but also the molecular mechanisms involved in gene regulation and ANs accumulation.

ANs are absorbed rapidly and were detected in the bloodstream and other tissues in different concentrations, both as ANs and as ANs-degradation metabolites. More investigations are required in this field, for a better overview and understanding of the chemical form and action association.

The potential of ANs to prevent and ameliorate several diseases such as diabetes, obesity, cancer, and cardiovascular and neuronal illnesses has been demonstrated in numerous in vitro and in vivo studies. Their antioxidant capacity and the inflammatory cytokines signaling are the key mechanisms of action that are believed to be responsible for such remarkable results.

Future ANs investigations and continued interest in ANs-based therapies will undoubtedly lead to new opportunities for pursuing the development of efficient formulations that would improve the ANs’ stability, bioavailability, and beneficial health effects.

## Figures and Tables

**Table 1:** In vitro studies of ANs showing their involvement in different pathologies such as diabetes, obesity, cancer, neurological, and cardiovascular.

	Cell Line	Source of ANs	Major ANs/ Metabolites	Effects	References
Diabetes and obesity	RAW264.7 (murine macrophages); 3T3-L1 (human preadipocyte)	Blackberry/ Blueberry beverages	Cy-3-glucoside	↓NF-ƙB ↓nitric oxide ↓TNF-α ↓isoproterenol-induced lipolysis ↓fat accumulation	[143]
	HepG2 (human hepatocellular carcinoma)	Blueberry extract Mv Mv-3-glucoside Mv-3-galactoside	Mv Mv-3-glucoside Mv-3-galactoside	↓ROS ↓lipogenesis/glycogenolysis enzymes ↑lipolysis via AMPK pathway	[144]
	D-HAEC (diabetic human aortic endothelial cells)	Bilberry/blueberry ANs capsules	Cy, Dp, Pt, Pn, Mv	↓NF-ƙB pathway ↓inflammatory and oxidative process	[145]
Cardiovascular diseases	HUVECs (human umbilical vein endothelial cells)	Standard solutions	Cy, Pn, Dp, 4-hydroxy- benzaldehyde	↓inflammatory and oxidative process ↓monocytes adhesion to HUVECs	[146]
		Red Chinese cabbage	Cy	↓TNF-α-induced NF-κB activity	[147]
		Blueberry juice	Protocatechuic acid Vanillic acid *trans*-ferulic acid *p*-coumaric acid	↑antioxidant response of Nrf2-regulated heme oxygenase 1 (HO-1) and glutamate-cysteine ligase modifier subunit (GCLM)	[148]
		Standard solutions	Cy-3-glucoside Pn-3-glucoside	↓levels of VCAM-1, ICAM-1, MMP-1, MMP-9 ↓activity of caspase-3, JNK, p38	[149]
	J774A.1 (murine monocyte macrophage)	*Hibiscus sabdariffa* L.	Dp, Cy	↓oxLDL ↓CD36 expression	[150]
Cancer	SMMC-7721 (human hepatoma cells)	*Lonicera caerulea* ‘Beilei’ fruit	Cy, Pn	↓cell proliferation ↑apoptosis	[151]
	HepG2 (human hepatocyte carcinoma)	Grape seeds	Pro-ANs	✓activation of caspase-3 ✓G2/M, G1/M cell cycle arrest	[152]
	DLD-1 SW480 SW620 Human colon cancer	Standard solution	Dp	↓tumor cells adhesion, migration, invasion, epithelial-to-mesenchymal transition ↓integrin and FAK signaling pathways ↑miR-204-3p upregulation	[153]
	HCT-29 HCT-116 Human colorectal cancer	Standard solutions	Dp-3-glucoside Cy-3-glucoside	↓PD-1, PD-L1	[154]
	Colon cancer stem cells	Purple fleshed potato extract	Pt, Mv, Cy, Pn	↓cell proliferation ↑cell apoptosis	[155]
	B16-F10 (murine melanoma cells)	Strawberry fruits	Cy, Pg, *p*-cumaroyl monohexose	↑cell differentiation ↓cell proliferation	[156]
	B16-F1 (murine melanoma cells)	Mulberry fruits	Cy, Pg	↓PI3K expression ↓Ras ↓NF-kβ	[157]
	MCF-7 HER2 MDA-MB-231 MDA-MB-453 Breast cancer cells	Black rice	Cy-3-glucoside Pn-3-glucoside	↓cell viability ↑caspase-3 activation ↑cytochrome C release	[158]
	MCF-7 (human breast cancer cells)	Red sorghum bran	-	↑apoptosis ↓tumor proliferation	[159]
	HeLa (human cervical tumor Cells)	Chokeberry	Cy-3-galactoside	✓antioxidant activity ↓cell proliferation	[160]
	MDA-MB-231 and MCF7 (human breast cancer cells)	Blueberry	Dp-3-glucoside Cy-3-glucoside Mv-3-glucoside	↓cell invasion capacity ✓activation of caspase-3 in MCF7 cells	[161]
	N202/1A, N202/1E (murine melanoma)	Strawberry	Pg-3-glucose	↓cell viability ✓ROS induction ✓mitochondrial damage	[162]
	B16-F10 (murine melanoma cells)	Elderberries	Cy-3-sambubioside-5-glucoside	↓cell proliferation ↑LDH activity	[163]
	Caco-2, HT-29 (colon cancer); MDA-MB-231 (breast cancer)	Table grapes with entacapone	Cy-3-glucoside Dp-3-glucose	↓cell proliferation ↑extracellular ROS levels	[164]
	MDA-MB-453 (breast cancer)	Black rice	Cy-3-glucoside Pn-3-glucoside	↓cell migration, adhesion, motility, invasion ↓urokinase-type plasminogen activator activity ↓transfer promoting factor activity	[165]
	MCF-7 (breast); SF-268 (CNS); NCI-H460 (lung); HCT-116 (colon); AGS (gastric) human tumor cells	Black/Red raspberry, Blackberry	Cy-3-glucoside Pg-3-glucoside Cy-3-glucosylrutinoside	↓cell proliferation ↓lipid peroxidation	[166]
	B16-F10 (murine melanoma)	Blueberry	Mv-3-galactoside Pt-3-galactoside Dp-3-galactoside	✓antioxidant activity ↓cells proliferation ✓apoptosis ↑LDH activity	[167]
Neurological disorders	SK-N-SH (human neuroblastoma) treated with Aβ25-35 (neurotoxic)	Standard solution	Cy-3-glucoside	↓ROS accumulation ↓ER stress response proteins ↓eIF2α, XBP-1, caspase-12	[168]
	SH-SY5Y (human neuroblastoma) treated with hydrogen peroxide (neurotoxic)	Blueberry/cranberry juices	Cy, Dp, Pn, Pt	↑SOD and CAT activity ↓ROS and TBARS accumulation	[169]

**Table 2:** In vivo studies of ANs and their health improvements in different pathologies such as diabetes, obesity, cancer, neurological, and cariological defects.

	Medical Condition	Source of ANs	Target Group	Treatment Details	Dose of Treatment	Effect	Reference
Diabetes and Obesity	Metabolic syndrome	Fresh bilberries	15 volunteers	8 weeks	400 g	↓IL-6, IL-12 ↓C-reactive protein ↓MMD and CCR2 expression	[186]
	Overweight/obesity	Black soybean extract	63 obese volunteers	8 weeks	2.5 g/day (ANs conc: 12.58 mg/g)	↓LDLc ↓TG ↓Non-HDLc	[187]
	Weight control over time	Blueberries Strawberries Apples Pears	124,086 volunteers	24 years	-	✓0.07–0.10 kg less weight gained every 4 years ✓weight control	[188]
	Overweight/obesity	Commercial red orange juice	11 women	12 weeks	500 mL/day	↓LDL	[189]
	Obesity	Raspberry extract	Male C57BL/6 mice	4 weeks	200 mg/kg food	↓TNFα, IL-6, NF-κB gene expression ↓63.7% less body weight ↑SOD, GSH-PX activity	[190]
	Obesity	Black rice Clack soybean Purple corn	C57BL/6 mice	12 weeks	200 mg/kg food	↓TNFα, IL-6, iNOS, NF-κB gene expression ↓lipid peroxidation ↑peroxide dismutase	[191]
	Obesity	Cherry Mulberry	C57BL/6 mice	8 weeks	200 mg/kg food	↓29.6 and 32.7% less body weight ↓TNFα, IL-6, iNOS, NF-κB gene ↑SOD, GPX activity	[192]
	Metabolic syndrome	Wild blueberries	Obese Zucker rats	8 weeks	8% of diet	↓IL-6, TNF-α, Nf-κB ↓C-reactive protein	[193]
	Diabetes	Cy-3-glucoside	KK-A(y) mice	5 weeks	0.2% of diet	↓RBP4 expression ↓blood glucose ↑Glut4	[194]
	Pre-diabetes	Kamchatka honeysuckle extract	24 Wistar rats	4 weeks	327 mg ANs/g	↑gut α and β glucosidase activity ✓ameliorates abnormal lipid/glucose metabolism	[195]
	Diabetic nephropathy	Purple corn extract	C57BLKS/J-Lepr^db^ mice	8 weeks	10 mg/kg BW	↓VEGF, HIF-1a ↓angiogenesis	[196]
	Pre-diabetes	Black currant extract	Sprague- Dawley rats	-	5 mg/kg BW	↑GLP-1	[197]
Cardiovascular diseases/Obesity	Myocardial infarction (MI)	ANs-rich fruits and vegetables	93,600 women, ages 25–42	18 years	-	↓MI risk	[198]
	Vascular impairments	Blueberry fruits	21 healthy men	1, 2, 4, 6 h after ingestion	319, 637, 766, 1278, and 1791 mg total	↑vascular function	[199]
	Cardiovascular risk	Strawberries	Healthy volunteers	1 month	500 g fruits/day	↓cholesterol ↓triglycerides ↓activated platelets ↑plasma antioxidant capacity	[200]
	Hypercholesterolemia	ANs mixture	150 volunteers	24 weeks	320 mg/day	↓hsCRP ↓sVCAM-1 ↓IL-1β	[201]
Cancer	Chronic B cell lymphocytic leukemia	Bilberry extract	30 patients	24 h	-	✓activation of caspase-3 ✓apoptosis of B CLL cells ↓Bcl-2/Bad pathway	[202]
	Induced melanoma	Dp pure solution	C57BL/6N mice	30 days	10 mg delphinidin/kg	↓melanoma-induced tumor growth	[203]
	UVB-mediated apoptosis	Dp	Female SKH-1 mice	1 and 8 h	1 mg/0.1 DMSO/mouse	↓apoptosis ↓cyclobutane pyrimidine dimers ↓8-OhdG ↓DNA damage	[204]
	UVB-induced inflammation	Cy-3-glucoside	Female SKH-1 mice	24 h	250 and 500 µM	↓COX-2, iNOS, PGE_2_, NF-κB ↓proinflammatory cytokines ↓p38 MAP kinase signaling	[205]
Neuroprotection	Dementia	Cherry juice	49 older adults (+70 years)	12 weeks	200 mL/day	↑cognition ↑speech fluency ↑short/long memory	[206]
	Cognitive degradation	Freeze-dried blueberries	37 older adults (60–75 years)	90 days	24 g/day	↓verbal errors ↓switch cost on task-switching test	[207]
	Cognition improvement	Freeze-dried wild blueberries	21 children (7–10 years)	1, 3, 6 h	15 or 30 g/day	↑cognitive performance	[208]
	Neuroinflammation mediated cognitive impairment	Korean black soybean	Male Sprague-Dawley rats	7 weeks	100 mg/kg ANs	✓memory improved ✓astrocytes and microglia activation ↓RAGE, BACE-1, Aβ expression	[209]
	Neuroinflammation	Korean black soybean	Male C57BL/6N mice	14 days	24 mg/kg/day	↓p-NF-κB, TNF-α, and IL-1β ↓cell apoptosis	[210]
	Alzheimer dementia	ANs	Male Wistar rats	-	200 mg/kg/day	↑SOD, CAT, GPX ↓ROS	[211]
	Age-related brain deficiency	Chokeberry extract	Male Kunming mice	8 weeks	15 or 30 mg/kg	↓COX2, TGF-β1 and IL-1 ↓DNA degradation	[212]

## Data Availability

Not applicable.

## Abbreviations

8-OhdG	8-hydroxydeoxyguanosine
Aβ	amyloid-β
ABC	ATP-binding cassette
AMPK	adenosine monophosphate-activated protein kinase
ANs	anthocyanins
ANS	anthocyanidin synthase
AOX1	aldehyde oxidase 1
ASE	accelerated solvent extraction
AVIs	anthocyanins vacuolar inclusions
BACE-1	beta site amyloid precursor protein cleaving enzyme 1
BBB	Blood–brain barrier
Bcl-2	B-cell lymphoma 2
BTL-like	bilitranslocase-like transporters
BW	body weight
CAT	catalase
CCL-2	C-C motif chemokine ligand 2
CCR2	C-C motif receptor 2
CDH4	cadherin 4
CHI	chalcone isomerase
CHS	chalcone synthase
CLDN14	claudin 14
COMT	catechol-O-methyltransferase
COX-2	cyclooxygenase-2
CPR	C-reactive protein
CRB3	Crumbs cell polarity complex component 3
Cy	cyanidin
CYP2E1	cytochrome P450 2E1
DFR	dihydroflavonol 4-reductase
D-HAEC	diabetic human aortic endothelial cells
Dp	delphinidin
EAE	enzyme-assisted extraction
eIF2α	eukaryotic initiation factor 2 α
ER	endoplasmic reticulum
F3H	flavanone 3-hydroxylase
F3′H	flavonoid 3′-hydroxylase
F3′5′H	flavonoid 3′,5′-hydroxylase
FAK	focal adhesion kinase
FMD	flow-mediated dilation
GCLM	glutamate-cysteine ligase modifier subunit
GLP-1	glucagon-like peptide-1
GLUT2	glucose transporter 2
GSH	glutathione
GSH-PX/GPX	glutathione peroxidase
GSTs	glutathione *S*-transferases
H_2_O_2_	hydrogen peroxide
HDLc	high-density lipoprotein cholesterol
HHPE	high hydrostatic pressure extraction
HIF-1a	hypoxia-inducible factor 1-alpha
HO•	hydroxyl radical
HO-1	heme oxygenase 1
hsCRP	high-sensitivity C-reactive protein
HUVECs	human umbilical vein endothelial cells
ICAM-1	intercellular adhesion molecule-1
IFN-γ	Interferon-gamma
IL	interleukin
iNOS	inducible nitric oxide synthase
JNK	c-Jun N-terminal kinase
LDH	lactate dehydrogenase
LED	light-emitting diodes
MAE	Microwave-assisted extraction
MAP	mitogen-activated protein
MATE	multidrug and toxic compound extrusion
MDA	melanoma differentiation-associated protein
MEF	moderate electric field
MMD	monocyte to macrophage differentiation-associated
MMP-1, MMP-2, MMP-9	matrix metallopeptidase 1, 2, 9
Mv	malvidin
MT	O-methyl transferase
NF-ƙB	nuclear factor kappa-B
Nrf2	nuclear factor erythroid 2-related factor 2
O_2_^−^	superoxide anion
oxLDL	oxidized low-density lipoprotein
PD-1, PD-L1	programmed death-1, -ligand 1
PEF	pulsed electric field-assisted extraction
Pg	pelargonidin
PGE_2_	prostaglandin E_2_
PI3K	phosphoinositide 3-kinase
PLE	pressurized liquid extraction
Pn	peonidin
Pt	petunidin
p-tau	hyperphosphorylated tau
RAGE	receptor for advanced glycation end products
RBP4	retinol binding protein 4
ROS	reactive oxygen species
RPE	retinal pigment epithelium
SFE	supercritical fluid extraction
SGLT1	sodium dependent glucose co-transporter 1
SNARE	soluble *N*-ethylmaleimide-sensitive factor attachment protein receptors
SOD	superoxide dismutase
TBARS	thiobarbituric acid reactive substances
TG	triglycerides
TNF	tumor necrosis factor
TXNIP	thioredoxin-interacting protein
UAE	Ultrasound-assisted extraction
UFGT	flavonoid 3-O-glucosyltransferase
UMAE	ultrasound/microwave-assisted extraction
uPA	urokinase plasminogen activator
sVCAM-1	soluble vascular cell adhesion molecule-1
VEGF	vascular endothelial growth factor
XBP-1	X-box binding protein 1
